# Predictors of non-adherence to antihypertensive medication in Kinshasa, Democratic Republic of Congo: a cross-sectional study

**DOI:** 10.1186/s13104-015-1519-8

**Published:** 2015-10-01

**Authors:** Aimée M. Lulebo, Paulin B. Mutombo, Mala A. Mapatano, Eric M. Mafuta, Patrick K. Kayembe, Lisa T. Ntumba, Alain N. Mayindu, Yves Coppieters

**Affiliations:** Department of Epidemiology and Bio-statistics, Faculty of Medicine, Kinshasa School of Public Health, University of Kinshasa, PO Box 11850, Kinshasa, DR Congo; Programme National Multisectoriel de Lutte contre le VIH, Ministère de la Santé, Kinshasa, DR Congo; Department of Epidemiology, Bio-statistics and Occupational Health, Mc Gill University, Montreal, QC Canada; Ecole de Santé Publique, Université libre de Bruxelles, Brussels, Belgium

**Keywords:** Hypertension, Antihypertensive drugs, Medication adherence, DRC

## Abstract

**Background:**

Hypertension remains a public health challenge worldwide. In the Democratic Republic of Congo, its prevalence has increased in the past three decades. Higher prevalence of poor blood pressure control and an increasing number of reported cases of complications due to hypertension have also been observed. It is well established that non-adherence to antihypertensive medication contributes to poor control of blood pressure. The aim of this study is to measure non-adherence to antihypertensive medication and to identify its predictors.

**Methods:**

A cross-sectional study was conducted at Kinshasa Primary Health-care network facilities from October to November 2013. A total of 395 hypertensive patients were included in the study. A structured interview was used to collect data. Adherence to medication was assessed using the Morisky Medication Scale. Covariates were defined according to the framework of the World Health Organization. Logistic regression was used to identify predictors of non-adherence.

**Results:**

A total of 395 patients participated in this study. The prevalence of non-adherence to antihypertensive medication and blood pressure control was 54.2 % (95 % CI 47.3–61.8) and 15.6 % (95 % CI 12.1–20.0), respectively. Poor knowledge of complications of hypertension (OR = 2.4; 95 % CI 1.4–4.4), unavailability of antihypertensive drugs in the healthcare facilities (OR = 2.8; 95 % CI 1.4–5.5), lack of hypertensive patients education in the healthcare facilities (OR = 1.7; 95 % CI 1.1–2.7), prior experience of medication side effects (OR = 2.2; 95 % CI 1.4–3.3), uncontrolled blood pressure (OR = 2.0; 95 % CI 1.1–3.9), and taking non-prescribed medications (OR = 2.2; 95 % CI 1.2–3.8) were associated with non-adherence to antihypertensive medication.

**Conclusion:**

This study identified predictors of non-adherence to antihypertensive medication. All predictors identified were modifiable. Interventional studies targeting these predictors for improving adherence are needed.

## Background

Hypertension (HTN) remains a public health challenge worldwide because of its high prevalence and related complications. In 2000, 26.4 % of the World’s adult population had hypertension [1]. In Sub-Saharan Africa (SSA), the prevalence of HTN varied from 6 to 48 % [2]. In the Democratic Republic of Congo (DRC), the prevalence of HTN has been increasing for the past three decades. In 1987, the prevalence was 14.2 % in urban and 9.9 % in rural areas [3] and almost doubled in 2005 to reach 26.7 % [4].

In SSA, the control of HTN is low. For example, in West African countries this control is low, less than 10 % except for Nigeria where it is approximately 30 % [5]. In the DRC, the Vitaraa study conducted in Southern Kivu found that 86.4 % of hypertensive patients were uncontrolled [6]. Uncontrolled HTN is one of the main cardiovascular risk factors (CVRF) [7] and based on the World Health Organization (WHO) estimates, at least 50 % of cardiovascular diseases (CVD) and 75 % of strokes are consequences of high blood pressure [8]. In the DRC, HTN-related complications including stroke, chronic kidney disease (CKD) are increasingly reported [9–11].

Non-adherence to antihypertensive medication (NAM) is one of the main factors contributing to uncontrolled HTN [12]. In fact, benefits of antihypertensive medication to decrease cardiovascular complications have been established. However, inadequate adherence to antihypertensive therapy or NAM has been linked to the development of cardiovascular complications. The NAM is described as a widespread problem in an insidious chronic condition like HTN [13] and studies have shown that populations of African descent with HTN were less adherent to medication compared with Caucasians. The overall prevalence of medication adherence in this population was lower for any definition used [14]. NAM is also associated with cardiovascular mortality risk. A cohort study conducted in Brazil reported that the risk of mortality from CVD was three times higher in patients discontinuing HTN medication compared with those who regularly took their medication [15]. Thus, given the situation described previously for the DRC, we believe that it is essential to identify and gain a better understanding of the factors associated with NAM in our settings with the overreaching goal to inform strategies for improving patient adherence to medication. Therefore, using the WHO framework that describes five dimensions of factors that may affect adherence to therapy and the Morisky Medication Scale, a validated self-reported medication scale, this study aims to measure non-adherence to antihypertensive medication and to identify its predictors.

## Methods

### Design

From October 2013 to November 2013, a cross-sectional study was carried out at Kinshasa Primary Health-care (KPHC) network facilities. This network consists of 51 facilities that allows for follow-up of patients with NCDs like hypertension and diabetes, living in Kinshasa [16].

### Study population

#### Inclusion criteria

We included in the study hypertensive patients >18 years who have been under antihypertensive drugs for at least a month.

#### Exclusion criteria

Hypertensive pregnant women were excluded.

### Sampling

Only 25, out of 51 facilities, scheduled patients’ visits during the survey were therefore selected. All patients present in the facility and who met the inclusion criteria were enrolled. The sample size was computed using the following formula $n \ge \frac{{Z_{\alpha }^{2} \cdot p \cdot q}}{{d^{2} }}$ where the **p** represents the proportion of non-adherence to antihypertensive medication (we assumed that p = 50 % because the proportion of NAM in the DRC is unknown), **q** (1 − p), **z** value of the standard normal distribution corresponding to a significance level of alpha of 0.05 (1.96) and **d** the precision degree that we assumed to 5 % too. The minimal size computed was 384 patients. A total of 408 patients present in the health facilities were selected but only 395 (96.8 %) were eligible and enrolled. The study protocol was approved by the institutional review boards of the Kinshasa School of Public Health, Bureau Diocésain des Oeuvres Médicales de Kinshasa, and Service Médical Armée du Salut and conducted in accordance with the Helsinki Declaration II. All study participants provided written informed consent.

### Data collection and study variables

Patients were surveyed using a structured questionnaire, either in French or translated in Lingala, the local language. Five surveyors had been trained over 3 days and the interview instrument was pretested prior to its use. Covariates were defined according to the WHO framework. This framework was used because actually, no model of medication adherence for HTN has been published. The Health Beliefs Model has been used in several studies but it does not include all the dimensions of the NAM factors like those described by the WHO framework [17]. The variables collected entailed social and economic characteristics of patients (gender, age, marital status, religious affiliation, education, employment); conditions-related variables (duration of HTN, control of blood pressure, experience of physical symptoms due to HTN, co-morbidity); therapy-related variables (duration of antihypertensive medication treatment, number of pills taken daily, experience of medication side effects, use of non-prescribed medication, treatment adherence); patients-related variables (knowledge of HTN risk factors and complications, knowledge of treatment benefit, perception of hypertension gravity and hypertension-related complications gravity, beliefs on BP medications importance, status acceptation); healthcare team and system-related variables (patient–provider relationship, patient education, perception of treatment cost, geographical accessibility, availability of antihypertensive drugs).

Co-morbidity has been defined in this study as HTN associated with diabetes mellitus (DM) or organ damage targets (heart disease, strokes, or CKD). Patients were interviewed if identified by a health professional as having one of these conditions.

Only the blood pressure measured by healthcare providers on the day of the survey and recorded in the patient medical file was considered. Three hundred ninety (390) out of three hundred ninety five (395) had the values of blood pressure recorded in their medical file (98.7 %). Uncontrolled HTN was defined, for a patient without co-morbidity, as a patient with a SBP ≥140 mmHg and a DBP ≥90 mmHg. For a patient with co-morbidity, uncontrolled HTN was defined as a patient with SBP ≥130 mmHg and for DBP ≥80 mmHg [18].

The self-reported medication adherence was measured using the Morisky Scale, which is a validated scale with a good internal consistency. Its Cronbach’s alpha has been reported to be of 0.90 in studies of inner-city patients with hypertension [19, 20]. Patients responded, “yes” or “no” to the four questions and have been categorized into three groups, namely high, medium and low adherence, as summarized in Table 1 [19]. Thereafter, adherence was dichotomized in order to facilitate statistical analysis. The low and medium adherence was merged and classified as non-adherent and patients with high adherence were classified as adherent, like previously used by Patel [21].

**Table 1:** Morisky Scale

High adherence, if the sum = 0; medium adherence, if the sum is comprised between 1 and 2 and low adherence if the sum is comprised between 3 and 4		
Have you ever forgotten to take your BP medicine?	0. No	1. Yes
Are you sometimes careless in regard to your medicine?	0. No	1. Yes
Do you skip your medicine when you are feeling well?	0. No	1. Yes
When you feel bad due to the medicine, do you skip it?	0. No	1. Yes

Source: Morisky et al. [19]

### Statistical analysis

All analyses were performed using Statistical Package for Social Sciences (SPSS) version 21.0. (SPSS, Inc., Chicago, IL, USA). Descriptive statistics were used to summarize the study population characteristics. Continuous variables were reported using mean with standard deviation. Means for age of non-adherent and adherent patients were compared using Student’s *t* test. Categorical variables were reported as a frequency and percentage and groups were compared using χ^2^ test. The logistic regression helped to identify independent predictors of non-adherence. All variables associated with non-adherence to antihypertensive medication in the bivariate analysis were included in the final model. Odds ratio (OR) with a corresponding 95 % confidence interval was reported to quantify the strength of association. Significance was set at p-value of less than 0.05.

## Results

### Patients’ socioeconomic and clinical characteristics

Tables 2 and 3 summarize socio-economic and clinical characteristics of the patients. A total of 395 patients were assessed. The mean age of participants was 63.3 ± 9.6 years and nearly 76 % were female; almost half were married (45.6 %) and Catholic Christians (48.9 %); 40.5 % completed at least secondary school and 63.5 % were unemployed.

**Table 2:** Socio-economic characteristics of hypertensive patients, Kinshasa, 2013 (n = 395)

Variables	n	%
Mean (SD), age	63.3 ± 9.6 years	
Female gender	300	75.9
Marital status		
Currently married	180	45.6
Widowed	178	45.1
Divorced/separated	24	6.1
Never married	13	3.3
Religious affiliation		
Catholic Christians	193	48.9
Protestant Christians	73	18.5
Pentecostal Christians	61	15.4
Kimbanguist Christians	24	6.1
Other religious affiliations	44	11.1
Education		
No formal	82	20.8
Primary	153	38.7
Secondary	126	31.9
Tertiary	34	8.6
Employment		
Unemployed/retired	174	44.0
Employed	144	36.5
Housewife	77	19.5

**Table 3:** Clinical characteristics of hypertensive patients, Kinshasa, 2013 (n = 395)

Variables	n	(%)
Duration of hypertension, year		
<5	220	55.7
≥5	149	36.7
Unknown	26	6.6
Duration of antihypertensive treatment, year		
<5	239	60.5
≥5	126	31.9
Unknown	30	7.6
Control of blood pressure, n = 390		
Uncontrolled	329	84.4
Controlled	61	15.6
Number of pills, n = 395		
Mean (SD)	1.7 (1.2)	
Experience of medication side effects	217	54.9
Experience of physical symptoms due to hypertension	255	64.6
Physical symptoms felt, n = 255		
Dizziness	157	61.6
Tiredness	84	32.9
Blurred vision	79	30.9
Headache	60	23.5
Palpitations	17	6.6
Additional symptoms	20	7.8
Presence of co-morbidity	213	53.9
Types of co-morbidity		
Diabetes	207	52.4
Heart diseases	6	1.5
Taking of non-prescribed medications	81	20.5
Adherence measurement		
Have you ever forgotten to take your BP medicine?	126	31.9
Are you sometimes careless in regard to your medicine?	84	23.8
Do you skip your medicine when you are feeling well?	124	31.4
When you feel bad due to the medicine, do you skip it?	87	22.0
Medication non-adherence	214	54.2

Approximately half of the patients suffered from HTN for less than 5 years. The mean systolic blood pressure was 148.6 ± 22.1 and 87.8 ± 14.3 mmHg for the diastolic blood pressure. The control of HTN was present in 15.6 % (95 % CI 12.1–20.0 %) patients. Almost two-thirds of the patients (64.6 %) experienced physical symptoms due to HTN. Dizziness was the most common physical symptom reported. Co-morbidities were present in 53.9 % of participants, of which DM was the most common (52.4 %).

More than half of the participants had less than 5 years of antihypertensive treatment duration. On average, the patients took 1.7 ± 1.2 pills daily. Approximately half of the patients (55.9 %) experienced medication side effects, 20.5 % declared taking non-prescribed medications. Around one in three patients reported to have forgotten to take blood pressure medicine (31.9 %) and to have skipped taking drugs when they felt well (31.4 %). Using the Morisky Scale, we found that over half of participants (54.2 %) were non-adherent to their medication.

### Patients’ knowledge, beliefs and perception

Table 4 summarizes the study of the population knowledge, beliefs and perception about HTN and its treatment. Poor knowledge of the risk factors related to lifestyle was observed. Stress/anxiety was the most frequent risk factors mentioned (67.6 %) and stroke was the most common complication mentioned (37.2 %). The control of blood pressure was the most common mentioned benefit of antihypertensive treatment (55.4 %), the majority of patients believed in the importance of blood pressure medication in controlling BP (86.3 %) and accepted their hypertensive status (85.6 %). Also, the majority of the patients in this study thought it possible to adhere to treatment (92.4 %).

**Table 4:** Knowledge and perception of hypertensive patients, Kinshasa, 2013 (n = 395)

Variables	n	(%)
Risk factors related to HTN mentioned		
Anxiety/stress	267	67.6
Do not know	61	15.4
Salt intake/unhealth diet	25	6.3
Alcohol intake	19	4.8
Other factors	28	7.1
Hypertension-related complications mentioned		
Stroke	147	37.2
Death	110	27.8
Heart diseases	22	5.6
Diabetes	14	3.5
Others	18	4.6
Knowledge of treatment benefit		
Control of BP	219	55.4
Decrease the risk of complications	105	26.6
Decrease the risk of mortality	46	11.6
Do not know	24	6.1
Healing	19	4.8
Beliefs that BP medications are helpful in controlling BP		
Very helpful/helpful	341	86.3
Somewhat helpful/not helpful	45	11.4
Do not know	10	2.3
Status acceptation	338	85.6
Self-perception of ability to adhere to treatment	365	92.4

### Health team and health system variables

Table 5 summarizes the health team and system variables and shows that almost all patients declared that their relationship with care providers was very good or good (94.7 %). More than half of the patients reported that their health facility provided hypertensive patients with education (65.1 %); 86.3 % declared they payed for treatment. Three-quarters of the patients thought that the cost of the treatment was inexpensive (73.3 %). The majority of the patients reported that antihypertensive drugs were available in the healthcare facility when they needed them (87.1 %). The healthcare facilities were for the most part accessible geographically (74.2 %).

**Table 5:** Health team and system related factors

Perception of patient-provider relationships	n	%
Very good/good	374	94.7
Somewhat good/bad	21	5.3
Patient education performed in the healthcare facility	257	65.1
Have paid for treatment	341	86.3
Perception of treatment cost, n = 341		
Very inexpensive/inexpensive	250	73.3
Very expensive/expensive	24	7.0
Do not know	67	19.6
Healthcare facility accessible geographically	293	74.2
Availability of antihypertensive medications in the healthcare facilities	343	87.0

### Predictors of non-adherence to antihypertensive treatment

Table 6 summarizes the predictors of NAM after logistic regression. Poor knowledge about complications of hypertension (OR = 2.4; 95 % CI 1.4–4.4); unavailability of antihypertensive drugs in the healthcare facilities (OR = 2.8; 95 % CI 1.4–5.5), lack of hypertensive patients education in the healthcare facilities (OR = 1.7; 95 % CI 1.1–2.7), prior experience of medication side effects (OR = 2.2; 95 % CI 1.4–3.3), uncontrolled blood pressure (OR = 2.0; 95 % CI 1.1–3.9) and taking of non-prescribed medications (OR = 2.2; 95 % CI 1.2–3.8) were independently associated with NAM in this study.

**Table 6:** Bivariate and multivariate analysis determinants of non-adherence to antihypertensive medication

Variables	Bivariate analysis		Multivariate analysis	
	Crude OR (95 % CI)	P value	Adjusted OR (95 % CI)	P value
Age		0.782	–	–
Gender	0.8 (0.5–1.3)	0.412	–	–
Education				
No formal	1.0 (0.5–2.3)	0.921	–	–
Primary	1.9 (0.9–4.2)	0.078	–	–
Secondary	1.5 (0.7–3.3)	0.272	–	–
Tertiary				
Occupation				
Unemployed/retired	0.9 (0.6–1.4)	0.832	–	–
Employed				
Duration of hypertension, year				
Unknown	0.6 (0.3–1.5)	0.317	–	–
≥5	1.3 (0.8–1.9)	0.284	–	–
<5				
Duration of antihypertensive treatment, year				
Unknown	0.9 (0.4–1.9)	0.897	–	–
≥5	1.2 (0.8–1.9)	0.342	–	–
<5				
Control of blood pressure n = 390				
Uncontrolled	1.8 (1.1–3.2)	0.032*	2.0 (1.1–3.7)	0.018*
Experience of medication side effects	1.9 (1.3–2.9)	0.001*	2.2 (1.4–3.3)	0.001*
Experience of physical symptoms due to hypertension	1.4 (0.9–2.1)	0.122	–	–
Presence of Co-morbidity	1.2 (0.8–1.7)	0.465	–	–
Taking of non-prescribed medications	1.7 (1.1–2.8)	0.042*	2.2 (1.2–3.8)	0.006*
Poor knowledge of risk factors related to HTN	0.7 (0.5–1.1)	0.140	–	–
Poor knowledge of HTN related complications	2.5 (1.4–4.3)	0.001*	2.4 (1.4–4.4)	0.003*
Poor knowledge of treatment benefit	1.5 (0.7–3.1)	0.324	–	–
Beliefs that BP medications are helpful in controlling BP	1.3 (0.7–2.3)	0.421	–	–
Accept hypertensive status	1.2 (0.7–2.0)	0.589	–	–
Perception of HTN gravity	1.1 (0.6–1.8)	0.838	–	–
Perception of HTN related complications gravity	1.2 (0.7–2.0)	0.526	–	–
Perception of patient-provider relationships	2.0 (0.8–4.9)	0.128	–	–
Lack of patient education performed in the healthcare facility	1.9 (1.3–2.9)	0.003*	1.7 (1.1–2.7)	0.023*
Perception of treatment cost	0.9 (0.6–1.6)	0.948	–	–
Healthcare facility accessible geographically	0.9 (0.6–1.5)	0.771	–	–
Unavailability of antihypertensive medications in the healthcare facilities	2.6 (1.3–5.2)	0.003*	2.8 (1.4–5.5)	0.003*

* p value <0.05

## Discussion

This study aimed to measure NAM and to identify its predictors. This study found a high prevalence of NAM and a poor control of blood pressure. Patients, therapy and health system factors were the predictors of NAM.

The prevalence of non-adherence to antihypertensive medication was high and consistent with previous findings [14, 22, 23]. However, this prevalence could have been underestimated because of two factors: age of the patients and level of the facilities surveyed. The mean age of patients in this study was 63.3 ± 9.6 years whereas a previous study conducted in the DRC reported a mean age of 54.3 ± 9.2 years among patients admitted to hospital for HTN-related complications [11]. Previous studies reported that, compared with older patients, younger patients were more likely to be non-adherent [24, 25]. Then, we posed a question on the follow-up of the younger hypertensive patients. Probably, younger hypertensive patients in Kinshasa are underdiagnosed. The Vitaraa Study conducted in Southern Kivu province of the DRC reported that more than half of the hypertensive patients were unaware of their status [6]. Given that older patients usually have several health concerns, the probability of HTN being diagnosed incidentally is high. Secondly, this study was carried out in primary healthcare facilities that use essential or generic drugs, which were inexpensive. The cost of drugs is described as a factor of non-adherence; it has been minimized in this study. Also, some non-adherent patients may have developed complications necessitating hospital admission at secondary and tertiary facilities. Overall, we believe that if this study was carried out at secondary and tertiary levels, the proportion of non-adherence would have been higher.

This study also reported a poor control of blood pressure. These findings corroborate previous studies carried out in African populations, where the proportion of blood pressure control was very low, less than 10 % [5]. This poor control may be explained partly by the presence of patients with co-morbidity at primary-level care facilities. Co-morbidity has been defined in this study as HTN associated with diabetes or organ damage targets. Hypertensive patients associated with these diseases are categorized as high cardiovascular risk and this category of patients cannot be followed at the primary-level care facilities [18]. The low proportion of blood pressure control reported in this study could explain the prevalence of HTN-related complications reported at secondary and tertiary levels care in the DRC.

This study reports poor knowledge about risk factors related to HTN. This has also been described by a previous study carried out in Pakistan [26]. Furthermore, stress is the main factor, as corroborated by a study carried out in Zimbabwe where 70.6 % of patients mentioned stress as a cause of hypertension [27]. However, risk factors related to lifestyle such as obesity, tobacco use and excessive alcohol consumption were not often mentioned. In this study, stroke has been the most mentioned as a complication of HTN in contrast with a study conducted in Pakistan, where the heart complication was the most mentioned (30.6 %) [24]. This difference may be explained by the fact that stroke has been described as a major CVD present in the black population [18].

Social and economic factors (education level, sex, age, employment) are described by the WHO as the NAM factors [14]. In this study none of these factors have been associated with NAM. We provided some explanations above on age and employment, the latter has not been associated with NAM, probably because the majority of patients declared that antihypertensive drugs were inexpensive.

Hypertension is considered as a condition without symptom, the experience of physical symptoms due to HTN influences treatment adherence significantly, patients who do not feel unwell may be less adherent. In a qualitative study carried out in Bandundu (a province of the DRC), hypertensive patients reported that they took medication only when they experienced perceived symptoms of HTN [28]. In this study, no association was found between patients experiencing physical symptoms and treatment adherence. This result is consistent with a previous study which did not find this association [29].

Patient education is described as a catalyst for lifestyle change and treatment adherence [27]. This association with treatment adherence has previously been reported [30]. Patient education improves the patient’s understanding of diseases, lifestyle change and medication. In this study, patients who admitted to benefitting from education and who had a good knowledge of hypertension and its complications respectively were twice more likely to be treatment adherent.

Also, this study found that patients who took non-prescribed medication were twice more likely to be non-adherent to treatment than other patients. We surmise that probably these patients use complementary and alternative medicine (CAM) which is defined as a “group of diverse medical and healthcare systems, practices, and products that are not presently considered to be part of conventional medicine” [31]. Previous studies reported an association between the use of CAM and treatment adherence and blood pressure control [32–35]. It is also reported that the experience of side effects is among the main predictors of CAM use. In this study, patients experiencing side effects were more non-adherent than their strong hypothesis of the use of CAM. Unfortunately, the use of CAM was not measured in this study, future studies may be carried out to assess the use of CAM in hypertensive patients in Kinshasa.

Unavailability of antihypertensive medication in healthcare facilities is a barrier to health care and is described as a factor of medication non-adherence. Patients who reported the availability of antihypertensive medication in healthcare facilities were about twice more likely to be adherent. These findings are consistent with that previously reported by Ghozzi in Tunis [36].

We acknowledge some limitations in this study. A potential selection bias may have been introduced in this study relating to the level of care facilities surveyed. Also, information bias was possible—firstly by using the self-reported questionnaire for measuring medication adherence and secondly by reporting the blood pressure measured by non standardized methods. However, these overall findings corroborate those found in previous studies. We think that the results of this study may be generalized to hypertensive patients living in Kinshasa.

## Conclusion

This is the first study in the DRC with a large sample to measure the antihypertensive non-adherence and to identify related predictors. These findings show that all predictors identified were modifiable by strengthening the capacity of healthcare providers by communication with patients. Antihypertensive medication should be made available in healthcare facilities.

## Abbreviations

BP: blood pressure
CAM: complementary and alternative medicine
CKD: chronic kidney disease
CVD: cardiovascular diseases
CVRF: cardiovascular risk factors
DM: diabetes mellitus
DRC: Democratic Republic of Congo
HTN: hypertension
KPHC: Kinshasa Primary Health-care
NAM: non-adherence to antihypertensive medication
NCDs: non communicable diseases
OR: odds ratio
SSA: Sub-Saharan Africa
WHO: World Health Organization
